# Identification of risk factors for infection after mitral valve surgery through machine learning approaches

**DOI:** 10.3389/fcvm.2023.1050698

**Published:** 2023-06-13

**Authors:** Ningjie Zhang, Kexin Fan, Hongwen Ji, Xianjun Ma, Jingyi Wu, Yuanshuai Huang, Xinhua Wang, Rong Gui, Bingyu Chen, Hui Zhang, Zugui Zhang, Xiufeng Zhang, Zheng Gong, Yongjun Wang

**Affiliations:** ^1^ Department of Blood Transfusion, The Second Xiangya Hospital, Central South University, Changsha, China; ^2^ Department of Laboratory Medicine, The Second Xiangya Hospital, Central South University, Changsha, China; ^3^ Department of Anesthesiology, Fuwai Hospital National Center for Cardiovascular Diseases, Chinese Academy of Medical Sciences, Peking Union Medical College, Beijing, China; ^4^ Department of Blood Transfusion, Qilu Hospital of Shandong University, Jinan, China; ^5^ Department of Transfusion, Xiamen Cardiovascular Hospital Xiamen University, Xiamen, China; ^6^ Department of Transfusion, The Affiliated Hospital of Southwest Medical University, Luzhou, China; ^7^ Department of Transfusion, Beijing Aerospace General Hospital, Beijing, China; ^8^ Department of Transfusion, The Third Xiangya Hospital, Central South University, Changsha, China; ^9^ Department of Transfusion, Zhejiang Provincial People's Hospital, Hangzhou, China; ^10^ Department of Basic Medical Sciences, Changsha Medical University, Changsha, China; ^11^ Institute for Research on Equity and Community Health, Christiana Care Health System, Newark, DE, United States; ^12^ Department of Respiratory Medicine, Second Affiliated Hospital of Hainan Medical University, Haikou, China; ^13^ Sino-Cellbiomed Institutes of Medical Cell & Pharmaceutical Proteins Qingdao University, Qingdao, Shandong, China; ^14^ Department of Basic Medicine, Xiangnan University, Chenzhou, China

**Keywords:** machine learning, cardiac valvular surgery, infection, random forest, LASSO, artificial network

## Abstract

**Background:**

Selecting features related to postoperative infection following cardiac surgery was highly valuable for effective intervention. We used machine learning methods to identify critical perioperative infection-related variables after mitral valve surgery and construct a prediction model.

**Methods:**

Participants comprised 1223 patients who underwent cardiac valvular surgery at eight large centers in China. The ninety-one demographic and perioperative parameters were collected. Random forest (RF) and least absolute shrinkage and selection operator (LASSO) techniques were used to identify postoperative infection-related variables; the Venn diagram determined overlapping variables. The following ML methods: random forest (RF), extreme gradient boosting (XGBoost), Support Vector Machine (SVM), Gradient Boosting Decision Tree (GBDT), AdaBoost, Naive Bayesian (NB), Logistic Regression (LogicR), Neural Networks (nnet) and artificial neural network (ANN) were developed to construct the models. We constructed receiver operating characteristic (ROC) curves and the area under the ROC curve (AUC) was calculated to evaluate model performance.

**Results:**

We identified 47 and 35 variables with RF and LASSO, respectively. Twenty-one overlapping variables were finally selected for model construction: age, weight, hospital stay, total red blood cell (RBC) and total fresh frozen plasma (FFP) transfusions, New York Heart Association (NYHA) class, preoperative creatinine, left ventricular ejection fraction (LVEF), RBC count, platelet (PLT) count, prothrombin time, intraoperative autologous blood, total output, total input, aortic cross-clamp (ACC) time, postoperative white blood cell (WBC) count, aspartate aminotransferase (AST), alanine aminotransferase (ALT), PLT count, hemoglobin (Hb), and LVEF. The prediction models for infection after mitral valve surgery were established based on these variables, and they all showed excellent discrimination performance in the test set (AUC > 0.79).

**Conclusions:**

Key features selected by machine learning methods can accurately predict infection after mitral valve surgery, guiding physicians in taking appropriate preventive measures and diminishing the infection risk.

## Introduction

Currently, more than one million heart disease patients worldwide undergo cardiac surgery annually (1). Additionally, with the aging of the population, senile valvular disease, coronary heart disease, and myocardial infarction caused by valvular disease are becoming increasingly common. Surgical treatments, such as prosthetic heart valve replacement or valve plasty, are radical treatments for severe heart valve disease (2, 3). Cardiac valvular surgery is a complex and time-consuming procedure, and postoperative infection is one of the common complications (4). Postoperative infections worsen the length of hospital stay and hospitalization costs, increase the need for antimicrobial therapy, increase mortality, and decrease the quality of life (5–8). Moreover, cardiac surgery is increasingly performed in older adults with more comorbidities. Thus, the incidence of postoperative infection is expected to increase unless preventive measures are improved.

The prediction of infection after cardiac surgery is complicated by its diverse causes. Many patients and surgical-related risk factors are associated with developing a postoperative infection. Although cardiac surgery is performed under aseptic conditions, incisions are susceptible to postoperative infection due to the long duration of surgery, prolonged use of mechanical ventilation, allogenic blood transfusions, open cavities, and indwelling catheter drainage. The current misuse of antibiotics in clinical practice has led to drug resistance in pathogenic bacteria, further promoting the development of infections (5, 9, 10). Moreover, although the relationships between several perioperative factors and postoperative infection risk have been investigated (11–13), many questions regarding the overall rate of postoperative infection development, potential risk factors, and effective preventive strategies remain unanswered. Therefore, finding key perioperative variables and predicting postoperative infection in patients undergoing surgery is greatly valuable in reducing postoperative infections.

In recent years, machine learning has been extensively applied in diagnostic imaging, electronic health record (EHR) exploitation, prediction models, and cancer prognosis (14, 15). Numerous research demonstrated that machine learning prediction models presented great accuracy for predicting postoperative complications (16, 17). Machine learning does not need to rely on researcher-selected features and linear dependencies; therefore, it has the potential to characterize better the complex interactions among risk factors (18). Although an increasing number of studies have identified perioperative variables that impact clinical outcomes (19), previous studies on risk prediction after cardiac surgery had relied primarily on traditional statistical methods, such as logistic regression or linear models, which typically focus on a relatively small number of clinical variables (20). Therefore, our study aimed to identify the critical factors related to postoperative infection after cardiac valvular surgery and establish a clinical prediction model for postoperative infection using machine learning methods.

## Materials and methods

### Data source and study design

This research was a retrospective observational study conducted between January 2016 and December 2018. Patients aged 18–75 years who underwent cardiac valvular surgery were recruited from different regions and different hospitals such as Fuwai Hospital National Center for Cardiovascular Diseases, Qilu Hospital of Shandong University, Affiliated Hospital of Southwest Medical University, Zhejiang Provincial People's Hospital, Xiamen Cardiovascular Hospital, Beijing Aerospace General Hospital, The Third Xiangya Hospital of Central South University, and The Second Xiangya Hospital of Central South University. We collected 27 mitral valve replacement cases from the Second Xiangya Hospital from January 2022 to September 2022 for external verification.

We enrolled patients who underwent mitral valvuloplasty, mitral valve replacement, and mitral valve replacement combined with tricuspid valvuloplasty. The exclusion criteria consisted of patients from had other cardiac surgery such as reoperative cardiac surgery, coronary artery bypass grafting, emergency surgery, or atrial septal defect, etc.; had a missing data rate of >80%; were infected within 30 days before surgery; had a hematological disease; or had active bleeding or multiple bleeding trauma were excluded.

This study focused on all infections occurring within 30 days postoperatively, including surgical site infections (SSIs) and infections occurring at other sites (e.g., pneumonia; cardiac device infection; urinary tract infection; mediastinitis; empyema; endocarditis; infectious myocarditis or pericarditis; Clostridium difficile colitis, and bloodstream infections). Patients with at least one infection were labeled “infection”, and those without infection were labeled “normal”.

This study was approved by the Third Xiangya Hospital's Medical Ethics Committee (NCT03885570).

### Data collection

The original clinical data were manually collected from EHR systems. A total of 91 perioperative variables were collected, including demographic data (gender, age, height, blood group, and weight), clinical characteristics (left ventricular dilatation, atrial fibrillation), perioperative laboratory indicators (RBC count, WBC count, Hb, hematocrit [Hct], PLT count, total protein, albumin, globulin, creatinine, prothrombin time [PT], ALT, AST, fibrinogen, LVEF, international normalized ratio [INR]) and, operation type, intraoperative data (cardiopulmonary bypass [CPB] precharge; minimum Hb/Hct/oxygen saturation; crystal/colloid bolus infusion volume; urine output; blood loss; machine blood; autologous blood; total input/output; operation time; CPB time; ACC time), concomitant disease (anemia, hypertension, diabetes, cerebrovascular disease), and other data (NYHA class, American Society of Anesthesiologists class). The preoperative variables were collected within 24 h before the day of surgery and the postoperative variables were collected occurred 48 h after the surgery.

We preprocessed and cleaned the raw data, including detecting typos and out-of-range values and imputing missing values. All variables with a missing-value rate of >20% were excluded; the remaining missing values were imputed using a predictive mean-matching imputation method.

Data were randomly divided, at a 70:30 ratio, into a training dataset (*n* = 858) and a testing dataset (*n* = 365).

### RF screening for important variables

The RF model for postoperative infection was generated using R packages (caret, Boruta, and randomForest) on the training dataset (*n* = 858). First, we assessed the mean model error rate for all variables according to out-of-band data. We set 49 as the optimal number of nodes and selected 436 as the optimal tree number in the RF. Then, we established the RF model and obtained the importance of each variable by the Gini coefficient method. We selected variables with an importance value greater than two for subsequent model construction.

### LASSO regression screening for important variables

Given LASSO regression's outstanding feature selection capabilities, we also performed LASSO regression on the training dataset (*n* = 858) and compared the results to those of the RF model in a Venn diagram. LASSO is a regression analysis method used for simultaneous feature selection and regularization. It adds an L1 norm as a penalty to calculate the minimum residual sum of squares. Tuning parameter (*λ*) selection in the LASSO model used 10-fold cross-validation *via* the minimum criteria. When *λ* is sufficiently large, some coefficients can be accurately reduced to zero. The curve of the binomial deviance was plotted depending on the log (*λ*). The dotted vertical lines represented the optimal value by adopting the minimum criteria with one standard error (1-SE criteria). The R package, glmnet, was used for LASSO regression.

### ML methods to build a diagnostic model

The ANN model was generated using the neuralnet R package on the training dataset (*n* = 858). Before training the neural network, we filtered and normalized the selected data by the min-max normalization method. The difference in each variable between the infected and noninfected groups was calculated. Then the selected data were assigned values of either 1 or 0 based on whether or not the variable's value was: >median with logFC > 0 or < median with logFC < 0. Additionally, we set the number of hidden layers to one and neurons to five. Accordingly, the selected variables were inputted into the ANN model, with one hidden layer with five neurons and two outputs (normal and infection). The infection classification score was calculated by multiplying the weight scores and the values of the important variables. Five-fold cross-validation of the model was performed using the R package, caret, and the confusion matrix function was adopted to evaluate model accuracy in the training (*n* = 858) and validation (*n* = 365) datasets. The termination condition was as follows: the error absolute partial derivative value was < 0.01. Eight other models were generated using the train function from the caret R package, and the models of SVM, LR, Random Forest, XGBoost, GBDT, AdaBoost, and Naive Bayes, nnet were developed and compared with the proposed machine learning model.

### Model performance evaluation

The AUC was used for the assessment of model performance. The AUCs of three types of scores (neural infection) were calculated for the training (*n* = 858) and validation (*n* = 365) datasets using the R package, pROC. The following assessment parameters were calculated: AUC, accuracy, sensitivity, specificity, positive predictive value, negative predictive value, and Balanced accuracy.

### Statistical analysis

Data analyses were performed using SPSS (IBM, Build 1.0.0.1126) and R software (version 4.0.4) with the abovementioned packages. Means and standard deviation (SD) were used to describe normally distributed data. Moreover, data were reported as the median and interquartile range (IQR) values for non-normally distributed data. For descriptive analyses, the Student's *t*-test or rank-sum test was used to evaluate differences in continuous variables between training and testing datasets. Fisher's exact test was used to evaluate differences in categorical variables. *P*-values < 0.05 were considered statistically significant.

## Results

### Study population and characteristics

**Figure 1:**
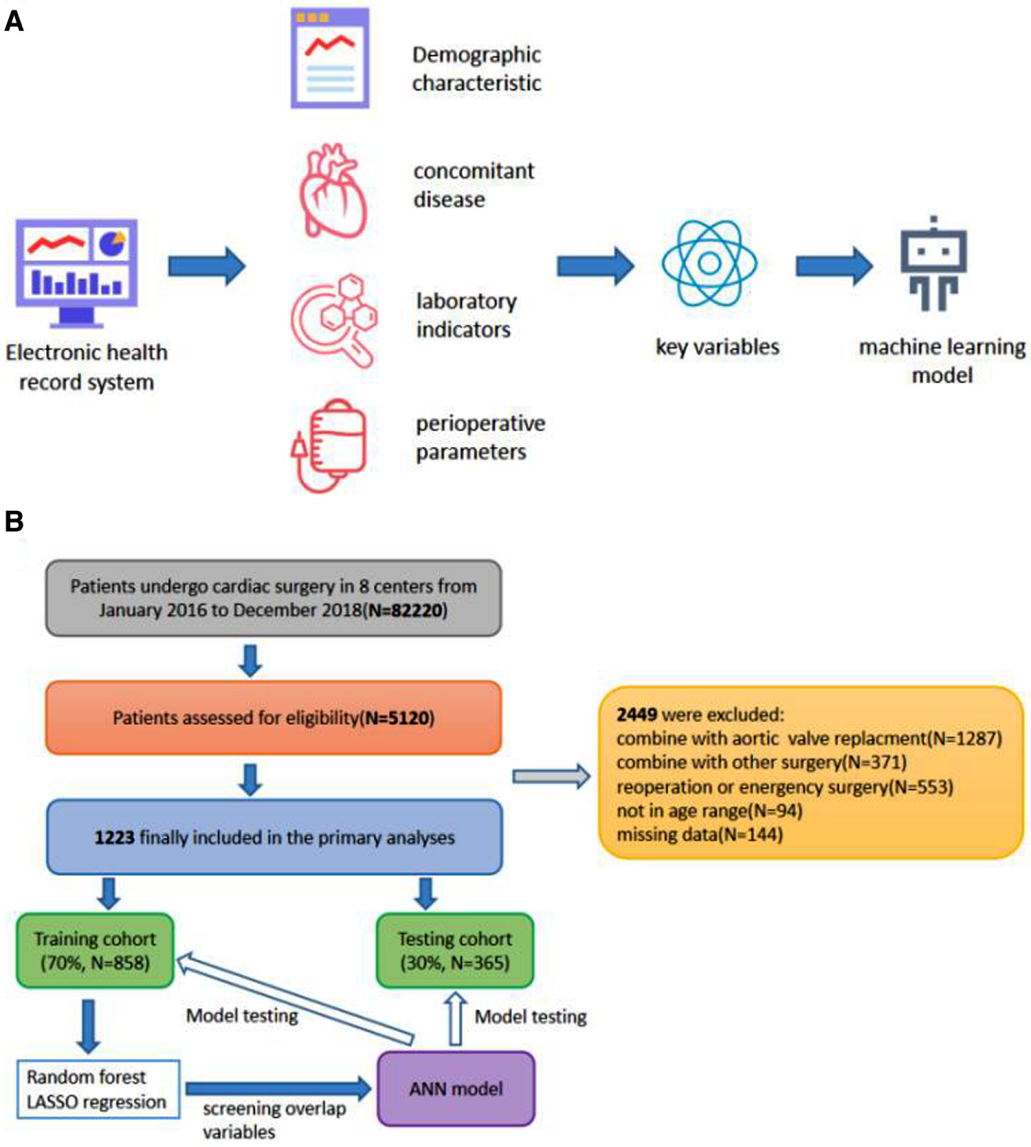
(**A)** Scheme showing the study design. (**B**) Flowchart of participant selection and procedure of the study.

Figure 1 shows the patient selection flowchart. The data of 82,220 patients treated between January 2016 and December 2018 were reviewed. After applying the study criteria, 1223 patients were included in the primary analyses. The baseline characteristics of the participants are presented in Table 1. The median age of the patients was 52.6 years. Men accounted for 39.9% of the study population, and the average body mass index was 22.9 kg/m^2^. Postoperative infections within 30 days after surgery occurred in 367 (30%) patients, including 15 (1.2%) patients with SSIs.

**Table 1:** Baseline characteristics and perioperative data of patients.

variables	Overall (*n* = 1,223)	Noninfection (*n* = 855)	Infection (*n* = 368)	*P*-value
Demographics characteristics				
Gender[male, *n* (%)]	488 (39.9)	366 (42.8)	122 (33.2)	0.002
Age, mean (SD)	52.6 (10.5)	52.6 (10.6)	52.5 (10.2)	0.887
Height [cm, mean (SD)]	160.5 (8.5)	161.4 (8.5)	158.9 (8.4)	<0.001
Weight [kg, mean (SD)]	60.2 (11.7)	61.1 (12.0)	58.0 (10.6)	<0.001
BMI, mean (SD)	22.9 (3.4)	22.9 (3.5)	22.9 (3.2)	0.883
Blood group, *n* (%)				0.013
A	389 (31.8)	266 (31.1)	123 (33.5)	
B	293 (24.0)	217 (25.4)	76 (20.7)	
O	433 (35.4)	286 (33.5)	147 (39.9)	
AB	108 (8.8)	86 (10.1)	22 (6.0)	
Comorbidities				
Atrial fibrillation, *n* (%)	638 (52.2)	428 (50.1)	210 (57.2)	0.024
LV dilatation, *n* (%)	566 (46.3)	344 (40.2)	222 (60.3)	<0.001
Hypertension, *n* (%)	190 (15.5)	145 (17.0)	45 (12.2)	0.039
Diabetes, *n* (%)	46 (3.8)	37 (4.3)	9 (2.4)	0.076
Cerebrovascular disease, *n* (%)	79 (6.5)	56 (6.5)	23 (6.3)	0.845
NYHA classification, *n* (%)				<0.001
Ⅰ	19 (1.6)	17 (2.0)	2 (0.5)	
Ⅱ	250 (20.5)	218 (25.5)	32 (8.7)	
Ⅲ	799 (65.3)	561 (65.6)	286 (77.8)	
Ⅳ	103 (8.4)	59 (6.9)	48 (13.0)	
ASA				<0.001
1	10 (0.8)	3 (0.4)	7 (1.9)	
2	56 (4.6)	38 (4.4)	18 (4.9)	
3	865 (70.8)	645 (75.4)	220 (59.8)	
4	292 (23.9)	169 (19.8)	123 (33.4)	
Presurgery Laboratory test				
RBC (10^12^/L), mean (SD)	4.5 (0.7)	4.6 (0.7)	4.3 (0.6)	<0.001
WBC (10^9^/L), median [Q1,Q3]	6.1 (5.0,7.5)	6.2 (5.1,7.6)	5.9 (4.9,7.3)	0.093
Hb, g/L, mean (SD)	130.2 (21.0)	131.3 (21.6)	127.7 (19.4)	0.006
Hct (/L), mean (SD)	40.5 (5.6)	40.8 (5.7)	39.7 (5.4)	0.001
PLT (10^9^/L), mean (SD)	202.9 (68.1)	205.6 (68.6)	196.7 (66.6)	0.037
Creatinine (µmol/L),median [Q1,Q3]	72.0 (61.0,85.7)	73.1 (61.7,85.6)	70.0 (59.9,85.9)	0.394
TP (g/L), mean (SD)	68.3 (6.7)	68.6 (6.9)	67.4 (6.2)	0.006
Albumin (g/L), mean (SD)	39.9 (4.5)	40.3 (4.8)	38.9 (3.8)	<0.001
Globulin (g/L), mean (SD)	28.7 (10.0)	28.4 (5.4)	29.4 (16.3)	0.101
ALT (IU/L), median [Q1,Q3]	19.7 (13.0,31.4)	20.0 (13.4,32.6)	18.8 (12.8,29.0)	0.126
AST (IU/L), median [Q1,Q3]	22.8 (18.0,29.4)	22.8 (18.0,29.6)	23.0 (18.8,29.0)	0.926
PT (s), median [Q1,Q3]	13.2 (12.1,14.5)	13.4 (12.5,14.6)	12.6 (11.6,14.2)	0.183
INR, median [Q1,Q3]	1.06 (1.0,1.2)	1.1 (1.0,1.2)	1.0 (1.0,1.2)	0.923
FIB (g/L), median [Q1,Q3]	2.9 (2.5,3.5)	3.0 (2.5,3.6)	2.8 (2.4,3.3)	0.001
Intraoperative information				
Charging time(min), median [Q1,Q3]	18.0 (14.0, 23.0)	18.0 (14.0,24.0)	17.0 (13.0,22.0)	0.177
Surgery time(min), mean (SD)	232.9 (61.0)	235.9 (57.6)	226.1 (67.7)	0.010
Blood loss op (ml), median [Q1,Q3]	600.0 (480.0, 600.0)	600.0 (400.0,600.0)	600.0 (600.0,600.0)	<0.001
CPB time (min), median [Q1,Q3]	94.0 (74.8, 188.0)	96.0 (77.0,125.0)	88.0 (68.0,109.3)	0.002
ACC time(min), median [Q1,Q3]	60.0 (44.0,81.0)	63.0 (48.0,84.0)	53.0 (37.0,71.0)	<0.001
Cardiopulmonary bypass precharge(ml), median [Q1,Q3]	1,600.0 (1,510.0,1,800.0)	1,600.0 (1,505.0,1,750.0)	1,750.0 (1,650.0,1,850.0)	<0.001
Total input op (ml), median [Q1,Q3]	2,896.0 (2,390.0, 3,600.0)	2,950.0 (2,410.0,3,790.0)	2,755.0 (2,350.0,3,250.0)	<0.001
Total output op (ml), median [Q1,Q3]	2,500.0 (887.0, 3,400.0)	2,100.0 (600.0,3,380.0)	2,800.0 (2,315.0,3,400.0)	<0.001
Autologous blood op (ml), median [Q1,Q3]	0 (0, 220)	160.0 (0,400.0)	0 (0,0)	<0.001
Total Blood tranfusion				
RBC(u), median [Q1,Q3]	2.0 (0,4.0)	1.0 (0,3.0)	2.0 (0,5.0)	<0.001
FFP(ml),median [Q1,Q3]	150.0 (0,400.0)	0 (0,400.0)	300.0 (0,450.0)	<0.001
PLT,median [Q1,Q3]	0 (0,0)	0 (0,0)	0 (0,0)	0.009
Cyoprecipitate(u),median [Q1,Q3]	0 (0,0)	0 (0,0)	0 (0,0)	<0.001

SD, standard deviation; BMI, body mass index; NYHA, New York Heart Association; ASA, The American Society of Anesthesiologists; RBC, red blood cell; WBC, white blood cell; Hb, hemoglobin; Hct, red blood cell volume; PLT, platelet; TP, total protein, ALT, alanine aminotransferase; AST, aspartate aminotransferase; PT, prothrombin time; INR, international normalized ratio; FIB, fibrinogen; LVEF, left ventricular ejection fractions; FFP, fresh frozen plasma; CPB, cardiopulmonary bypass precharge.

### Feature selection by RF modeling

**Figure 2:**
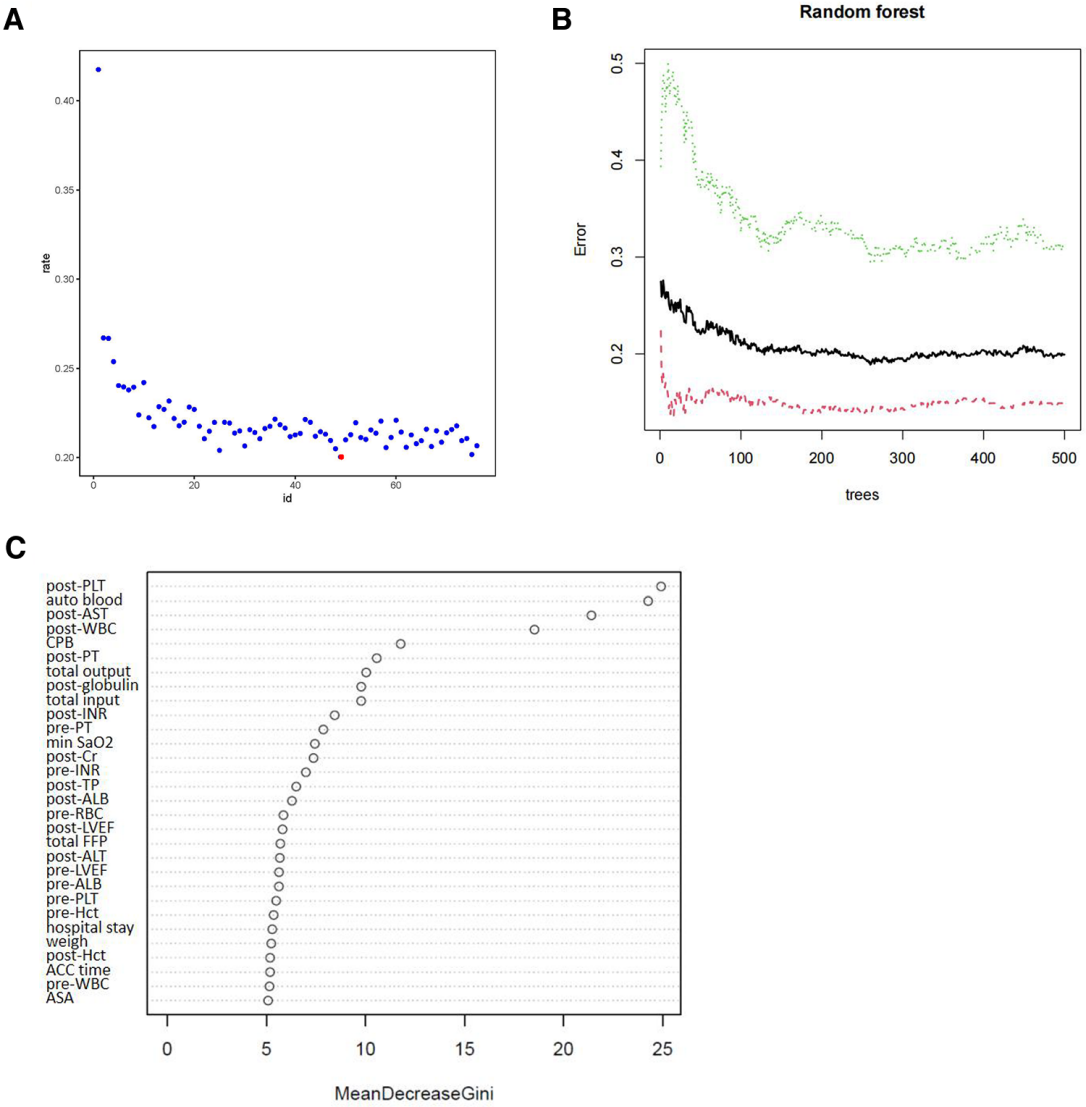
Random forest analysis was performed to screen candidate variables. (**A**) The scatter plot of the variables. The y-axis represents the out-of-band error rate, and the x-axis shows the variables’ number. The red point represents the optimal number of variables (47). (**B**) The number of decision trees according to the error rate. The y-axis represents the error rate, and the x-axis represents the number of decision trees. (**C**) Variables were sorted with the Gini importance parameter in the random forest model. The top 30 variables are listed based on Mean Decrease Gini.

These patients were randomly divided into a training set ((*n* = 858) and a testing dataset (*n* = 365) (Table 2). The results revealed that the *p*-values of variables for the training and testing sets were greater than 0.05, indicating no significant differences between training and test dataset variables. Figure 2 showed the training process and optimal parameters of the RF model. The average error rate when all features were selected is shown in Figure 2A. Keeping the variables number and the out-of-band error minimized as much as possible, we selected 47 as the number of variables (Supplementary File S1). According to the correlation map between the number of decision trees and the model error (Figure 2B), we chose 436 trees as the final model condition, which showed the lowest error rate. The 47 variables with an importance score above 2 were selected as specific variables for further model construction. Figure 2C presents the importance matrix plot of the top 30 variables. Postoperative PLT and intraoperative autologous blood were the most important factors, followed by postoperative AST and postoperative WBC count.

**Table 2:** Baseline characteristics and perioperative data in model training and testing cohorts.

Variables	Overall (*n* = 1,223)	Training cohort (*n* = 858)	Testing cohort (*n* = 365)	*P*-value
Demographics characteristics				
Gender[male, *n* (%)]	488 (39.9)	343 (40.0)	145 (39.7)	0.953
Age, mean (SD)	52.6 (10.5)	52.7 (10.4)	52.2 (10.6)	0.467
Height [cm, mean (SD)]	160.5 (8.5)	160.6 (8.6)	160.3 (8.5)	0.611
Weight [kg, mean (SD)]	60.2 (11.7)	60.3 (11.9)	59.9 (11.3)	0.567
BMI, mean (SD)	22.9 (3.4)	22.9 (3.4)	22.9 (3.4)	0.806
Blood group, *n* (%)				0.831
A	389 (31.8)	272 (31.7)	117 (32.1)	
B	293 (24.0)	210 (24.5)	83 (22.7)	
O	433 (35.4)	298 (34.7)	135 (37.0)	
AB	108 (8.8)	78 (9.1)	30 (8.2)	
Comorbidities				
Atrial fibrillation, *n* (%)	638 (52.2)	448 (52.2)	190 (52.1)	0.944
LV dilatation, *n* (%)	566 (46.3)	391 (45.6)	175 (47.9)	0.456
Hypertension, *n* (%)	190 (15.5)	130 (15.2)	60 (16.5)	0.875
Diabetes, *n* (%)	46 (3.8)	31 (3.6)	15 (4.1)	0.832
Cerebrovascular disease, *n* (%)	79 (6.5)	59 (6.9)	20 (5.5)	0.361
NYHA classification, *n* (%)				0.939
Ⅰ	19 (1.6)	15 (1.7)	4 (1.1)	
Ⅱ	298 (24.3)	213 (24.8)	72 (19.7)	
Ⅲ	803 (65.6)	558 (65.0)	244 (66.8)	
Ⅳ	103 (8.4)	72 (8.4)	31 (8.5)	
ASA, *n* (%)				0.925
1	10 (0.8)	7 (0.8)	3 (0.8)	
2	56 (4.6)	37 (4.3)	19 (5.2)	
3	865 (70.7)	609 (71.0)	256 (70.1)	
4	292 (23.9)	205 (23.9)	87 (23.8)	
Presurgery Laboratory test				
RBC (10^12^/L), mean (SD)	4.5 (0.7)	4.5 (0.7)	4.5 (0.7)	0.226
WBC(10^9^/L), median [Q1,Q3]	6.1 (5.0,7.5)	6.1 (5.0,7.5)	6.2 (5.1,7.6)	0.408
Hb, g/L, mean (SD)	130.2 (21.0)	130.3 (20.7)	129.9 (21.9)	0.766
Hct(/L), mean (SD)	40.5 (5.6)	40.6 (5.5)	40.3 (5.8)	0.474
PLT (10^9^/L), mean (SD)	202.9 (68.1)	200.2 (66.9)	209.3 (70.4)	0.031
Creatinine(µmol/L),median [Q1,Q3]	72.0 (61.0,85.7)	72.0 (61.0,86.3)	71.8 (61.2,85.1)	0.846
TP (g/L), mean (SD)	68.3 (6.7)	68.3 (6.9)	68.2 (6.3)	0.767
Albumin (g/L), mean (SD)	39.9 (4.5)	40.0 (4.5)	39.7 (4.6)	0.377
Globulin (g/L), mean (SD)	28.7 (10.0)	28.4 (5.2)	29.3 (16.4)	0.160
ALT (IU/L), median [Q1,Q3]	19.7 (13.0,31.4)	19.3 (13.0,30.5)	20.3 (13.0,32.8)	0.957
AST (IU/L), median [Q1,Q3]	22.8 (18.0,29.4)	22.8 (18.0,29.0)	23.0 (18.0,30.0)	0.888
PT (s), median [Q1,Q3]	13.2 (12.1,14.5)	13.2 (12.1,14.5)	13.1 (12.0,14.4)	0.139
INR, median [Q1,Q3]	1.06 (1.0,1.2)	1.07 (1.0,1.2)	1.06 (1.0,1.2)	0.316
FIB (g/L), median [Q1,Q3]	2.9 (2.5,3.5)	2.9 (2.4,3.5)	3.0 (2.5,3.6)	0.128
Intraoperative information				
Charging time(min), median [Q1,Q3]	18.0 (14.0, 23.0)	17.0 (14.0,23.0)	19.0 (14.0,26.0)	0.007
Surgery time(min), mean (SD)	232.9 (61.0)	231.6 (58.1)	236.0 (67.1)	0.247
Blood loss op (ml), median [Q1,Q3]	600.0 (480.0, 600.0)	600.0 (480.0, 600.0)	600.0 (485.0, 600.0)	0.447
CPB time (min), median [Q1,Q3]	94.0 (74.8, 118.0)	93.0 (75.0,118.0)	94.0 (73.2, 120.8)	0.863
Aortic cross clamp time(min), median [Q1,Q3]	60.0 (44.0,81.0)	60.0 (44.0,80.0)	59.0 (44.0,82.0)	0.488
Cardiopulmonary bypass precharge(ml), median [Q1,Q3]	1,600.0 (1,510.0,1,800.0)	1,600.0 (1,510.0,1,800.0)	1,600.0 (1,510.0,1,800.0)	0.978
Total input op (ml), median [Q1,Q3]	2,896.0 (2,390.0, 3,600.0)	2,880.0 (2,368.0,3,548.5)	2,930.0 (2,430.0,3,750.0)	0.045
Total output op (ml), median [Q1,Q3]	2,500.0 (887.0, 3,400.0)	2,500.0 (800.0,3,300.0)	2,500.0 (1,000.0,3,600.0)	0.021
Autologous blood op (ml), median [Q1,Q3]	0 (0, 220)	0 (0, 212.3)	0 (0, 240)	0.640
Total Blood tranfusion				
RBC(u), median [Q1,Q3]	2.0 (0,4.0)	2.0 (0,4.0)	2.0 (0,4.0)	0.256
FFP(ml),median [Q1,Q3]	150.0 (0,400.0)	165.0 (0,400.0)	150.0 (0,400.0)	0.179
PLT,median [Q1,Q3]	0 (0,0)	0 (0,0)	0 (0,0)	0.924
Cyoprecipitate(u),median [Q1,Q3]	0 (0,0)	0 (0,0)	0 (0,0)	0.401

### Feature selection by LASSO regression

**Figure 3:**
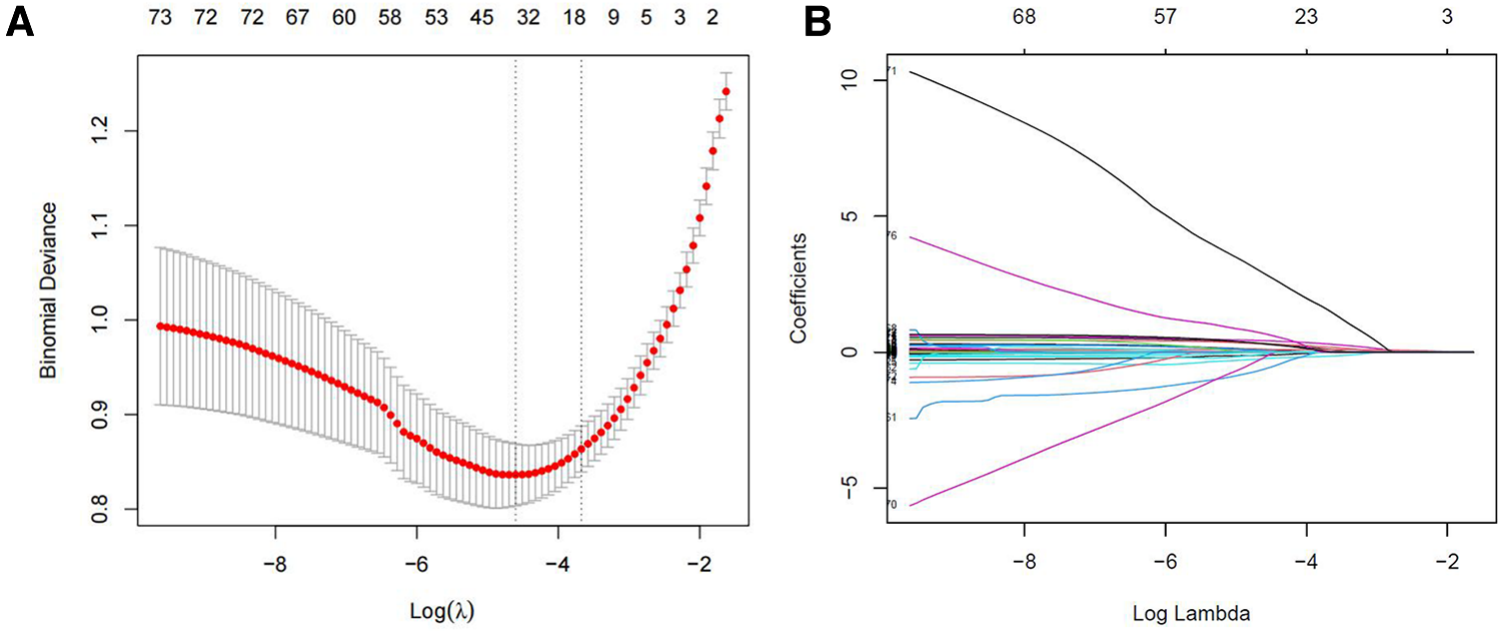
Feature selection using the LASSO regression. (**A**) Cross validation plot for the penalty term. (**B**) The coefficients of each predictor when 91 variables were included in the LASSO regression model.

Figure 3 showed the process and results of feature selection using LASSO. An optimal *λ* of 0.01000498 and log (*λ*) of −4.604672 were selected (1-SE criteria) according to 10-fold cross-validation and adopted in the LASSO regression. As shown in Figure 3A, the 91 features were finally decreased to 35 when using the above parameters. Figure 3B showed the LASSO coefficient profiles of the 91 features, plotted against the log (*λ*) sequence. A vertical line was drawn at the value selected using 10-fold cross-validation, resulting in 35 features with nonzero coefficients (Supplementary File S1).

### Construction of the prediction model

**Figure 4:**
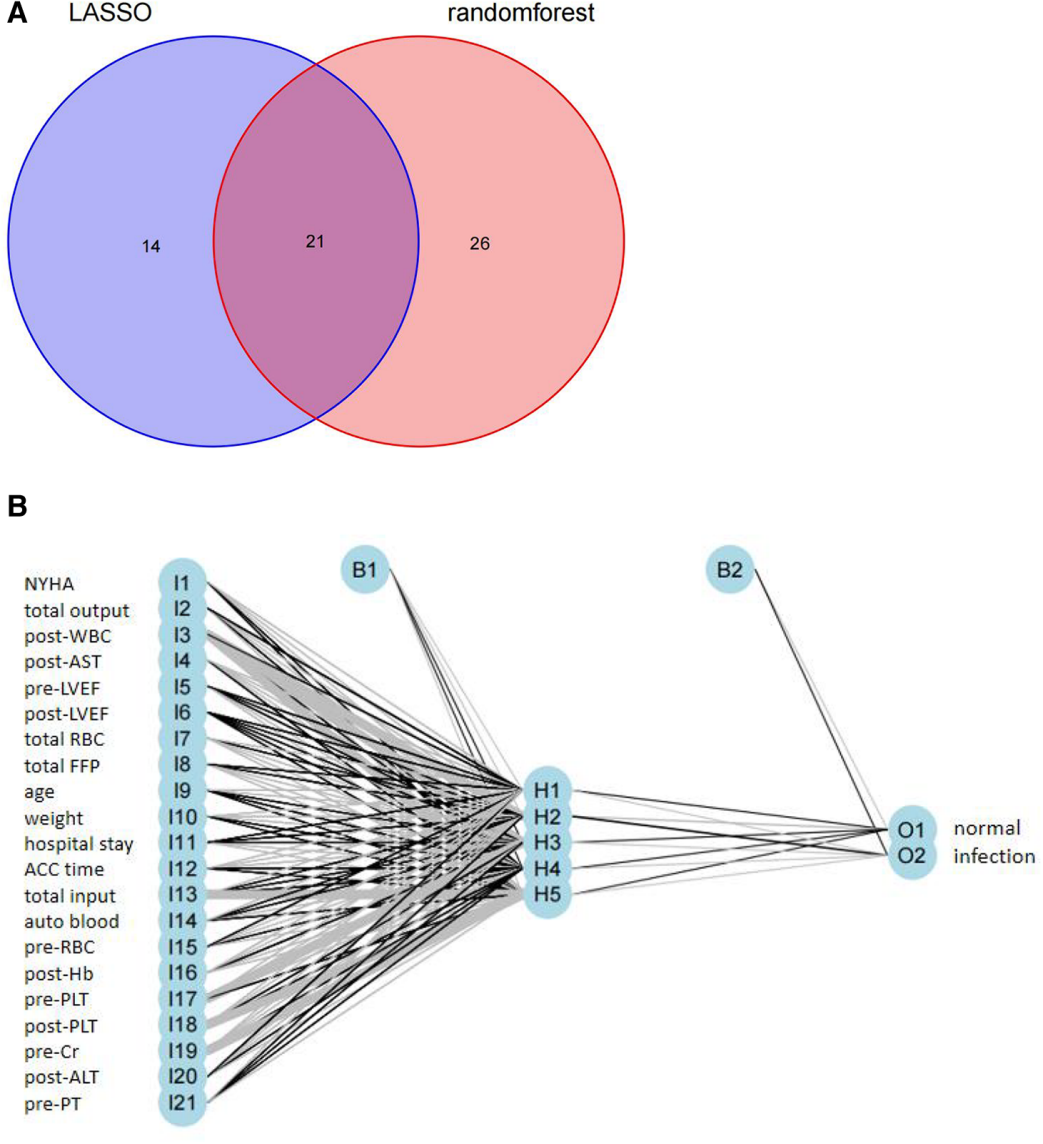
(**A)** Venn diagram showing the overlap between the variables were selected by the RF and the variables were selected by the LASSO; (**B**) results of neural network visualization.

**Figure 5:**
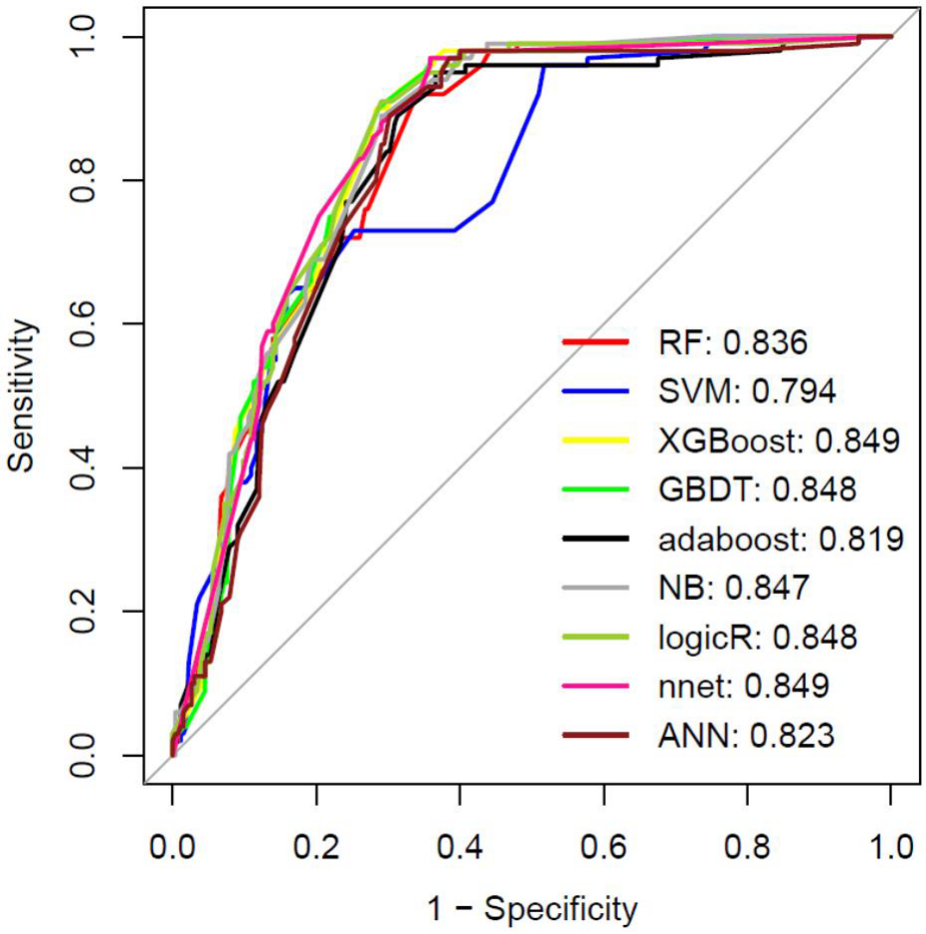
The machine learning models performance evaluation and prediction. The ROC result in the testing dataset. RF: Random Forest; SVM, Support Vector Machine; XGBOOST, extremely Gradient Boosting; GBDT: Gradient Boosting Decison Tree; NB: Naive Bayesian; LogicR: Logistic Regression; nnet: Neural Networks.

The intersection of the RF and LASSO results was shown in a Venn diagram in Figure 4A. We identified 21 overlapping important features, including age, weight, length of the hospital stay, total RBC transfusion, total FFP transfusion, and six preoperative factors (RBC count, PLT, NYHA class, LVEF, Cr, and PT), four intraoperative factors (autologous blood, ACC time, total input, and total output), and six early postoperative factors (PLT, AST, ALT, Hb, LVEF, and WBC count). We performed a correlation analysis between these features (Supplementary File S2). We could see that the highly correlated variables in the heat map do not appear in the final selected variables. Then, we compared the different models (ANN, RF, SVM, XGBoost, GBDT, NB, Adaboost, LogicBag, or Nnet) performance on the testing dataset, and the results indicated all the ML model showed excellent discrimination performance, the AUC value was ranged from 0.794 to 0.849. (Figure 5). As described in Table 3, the ACC, sensitivity, specificity, and BACC of the 9 models were 0.6822 ∼ 0.7836, 0.2600 ∼ 0.5700, 0.8302 ∼ 0.9245, and 0.5508 ∼ 0.7058, respectively.

**Table 3:** Model selection results for all machine learning models.

Method	AUC	Accuracy	Sensitivity	Specificity	BACC
RF	0.836	0.7,836	0.5,200	0.8,330	0.7,015
SVM	0.794	0.7,726	0.5,200	0.8,679	0.6,940
XGBoost	0.849	0.7,808	0.5,400	0.8,717	0.7,058
GBDT	0.848	0.7,808	0.5,400	0.8,717	0.7,058
Adaboost	0.819	0.7,808	0.5,400	0.8,717	0.7,058
NB	0.847	0.7,616	0.3,300	0.9,245	0.6,273
LogicR	0.848	0.6,822	0.2,600	0.8,415	0.5,508
nnet	0.849	0.7,781	0.5,400	0.8,679	0.7,040
ANN	0.823	0.7,589	0.5,700	0.8,302	0.7,001

Although the predicted result of the ANN model had no obvious superiority, we observed that the ANN model with the top *P* value (*P* = 0.9151) after McNemar's Test (Supplementary File S3), which indicated that the ANN predicted result could more correctly reflect the actual situation in the test set. The ANN model was conducted for our detailed analysis. Considering there is no fixed rule for the number of layers and neurons for parameter selection and the optimal number of hidden layer neurons should be situated in the number between output and input layer sizes, we set the number of hidden layers to 1 and neurons to 5. In Figure 4B, we used the five-fold cross-validation to evaluate the classification model performance. The following assessment parameters: accuracy, sensitivity, specificity, positive predictive value, and negative predictive value were shown in Supplementary File S4.

### ANN model performance and external validation

The AUC of the ANN model was 0.823 in the testing dataset (Figure 5). The AUC was 0.818 in the external verification dataset (Supplementary File S5). Thus, the ANN model showed good performance. The parameters of the ANN model were shown in Supplementary File S6. The validation curve for our ANN model based on using the different number of hidden neurons parameter was shown in Supplementary File S7. The AUC value of test set stabilized between 0.7 and 0.95. It indicated that our ANN model had good generalization capacities to prevent overfitting. Furthermore, we calculated the AUC for each variable to evaluate the selected variables possibly influencing the risk for the outcome (Supplementary File S8).

### Machine learning models for SSI

**Figure 6:**
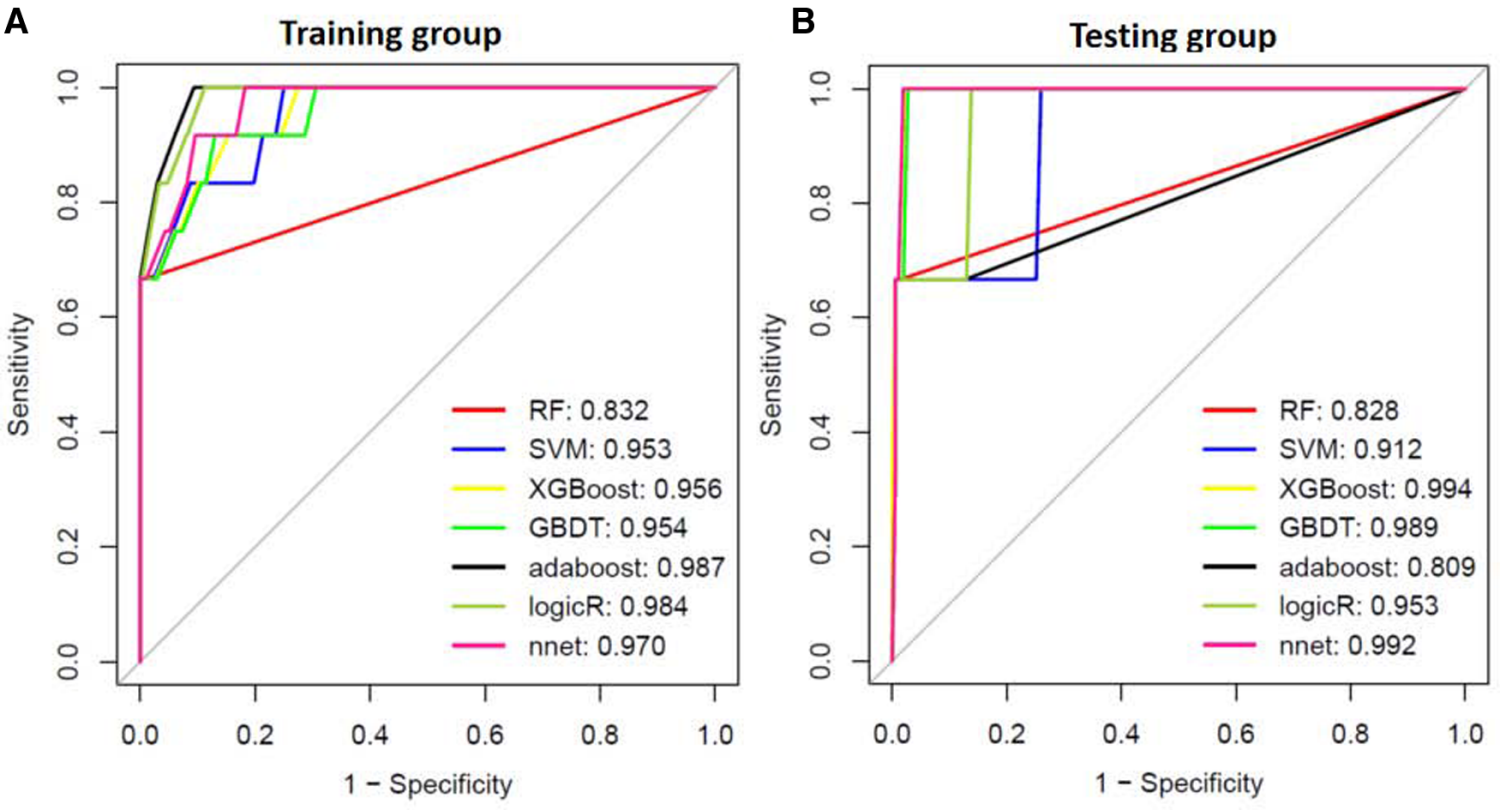
The area under the ROC curve for surgical site infection(SSI) for ML models.

We also generated prediction models based on our identified multiple variables for SSI. As shown in Figure 6, The least AUC of the seven machine learning models for SSI was >0.832 in the training dataset and >0.809 in the testing dataset.

## Discussion

In this study, we collected data from eight medical centers to identify critical variables associated with infection after cardiac surgery, based on the intersection of variables selected by RF modeling and LASSO regression, to construct a prediction model that could accurately predict infection after mitral valve surgery. The prediction models for infection after mitral valve surgery were established based on these variables, and they all showed excellent discrimination performance in the testing dataset (AUC > 0.79). The developed ANN model resulted in AUC scores of 0.875, 0.823, and 0.818 for the training, testing, and external verification datasets, respectively. Thus, the risk factors selected by machine learning methods can accurately predict infection after mitral valve surgery.

In the present study, the infection rate after cardiac surgery was 30.0%, higher than that in recently reported cardiac surgery cohorts (13.3%–20.3%) (21–23). However, we included all infections, including SSIs and other infections (pneumonia, bloodstream infections, deep sternal infections, and urinary tract infections). The aging of the population and the increase in postoperative invasive procedures in recent years might be another reason for the difference in infection rates. The rate of SSIs in this study (1.2%) was similar to that reported by other centers (5, 24).

Despite advances in surgical techniques, sterilization, asepsis, and antibiotic prophylaxis, infections complicate many patients' postoperative course (25). The factors influencing the risk of postoperative infection in heart valve surgery are complex. Previous studies have reported the procedure duration, age, number of blood transfusions, smoking history, and comorbid disease as risk factors for infection (26). As EHR systems provide a large amount of patient data, novel associations between specific perioperative variables and postoperative complications will likely be identified. However, the main difficulty in constructing a predictive model using EHR data is identifying the most critical variables or features.

Applying machine learning algorithms for clinical data analysis has revolutionized cardiovascular research methods. Recent research has shown that machine learning algorithms outperform traditional statistical modeling approaches. The RF and LASSO methods were the most widely used machine learning methods for feature selection in most literature (27–29). Especially the LASSO method might help to solve the collinearity problem. The unusual methods often cause overfitting in many datasets, making the results hard or impossible to repeat in another dataset. So we think this research adopting the widely used RF and LASSO would be helpful to obtain more practical clinical value results. As a classic machine learning algorithm, RF modeling has high accuracy in disease risk prediction and diagnosis. We calculated the importance of each variable to postoperative infection using RF modeling and visualized its contribution. LASSO regression, as a type of linear regression, performs well in reducing the data dimensions and multicollinearity among features, and it is generally used in predictive models to select meaningful feature values among a large number of variables. The present study used RF modeling and LASSO regression to identify critical variables related to postoperative infection. The intersection of variables screened separately by RF modeling and LASSO regression was determined using a Venn diagram. We identified 21 key variables, including age, weight, length of the hospital stay, total RBC transfusion, total FFP transfusion, six preoperative factors (RBC count, PLT, NYHA class, LVEF, creatine, and PT), four intraoperative factors (autologous blood, ACC time, total input, and total output), and six early postoperative factors (PLT, AST, ALT, Hb, LVEF, and WBC count). Among these variables, some were known risk factors (e.g., age, weight, length of hospital stay, and allo-blood transfusions), and some were previously unreported predictors.

The preoperative factors identified in the present study were mainly related to cardiac function and coagulation indicators. Clinicians can pay attention to RBC count, PLT, NYHA class, LVEF, PT, and other indicators when the patient is admitted to the hospital, and focus on improving the patient's anemia, coagulation function, and heart function during the treatment. Intraoperative total input and total output are important volume indicators reflecting the acute physiological responses during surgery and play critical roles in the development of infection. Interestingly, we found that intraoperative autologous blood transfusion was strongly related to postoperative infection. A previous study showed that autologous blood transfusion reduces the transfusion of allogeneic blood components (30). However, a meta-analysis found that autologous blood transfusion during cardiac surgery was not associated with less postoperative infection (31). Additionally, Jan et al. reported that cell salvage is directly associated with a higher infection rate (32). The direct effect of autologous blood transfusion on postoperative infection risk has not been previously demonstrated, and the mechanisms may require further exploration. For intraoperative risk factors, clinicians can improve the operation method to shorten the ACC time, minimize the patient's blood loss, and strictly control the intake.

Laboratory biomarkers in the early postoperative period can reflect the acute pathophysiology of infection. In the present study, we identified six laboratory indicators associated with infection. The number of WBCs in peripheral blood directly reflects the inflammation level in the body. The elevated white blood cell (WBC) count has traditionally been a predictor of infection in clinical practice. Recently several studies also reported that increased preoperative WBC count is an independent predictor of postoperative cardiac infection (33). And the literature reported that the combination of PCT and WBC levels over the first 3 postoperative days was able to predict postoperative infection within the 30 days following cardiac surgery (34). The postoperative WBC count in our study was collected early (within 48 h after the surgery) and we excluded patients who were infected within 30 days before surgery. Thus, the result indicated that early postoperative WBC count was a predicted indicator of postoperative infection. PLT and AST levels also reflect the severity of the patient's condition and are closely associated with infection (35–37). For early postoperative risk factors, clinicians should pay special attention to PLT, AST, ALT, Hb, LVEF, and WBC count within 48 h after surgery. These important perioperative factors may help guide individualized preventive strategies and aid in proper infection management after cardiac surgery.

A strength of the present study is that we evaluated the risk factors of various postoperative infections within a sizeable multicenter cohort, rendering our results generalizable to patients undergoing mitral valve surgery. Furthermore, the ANN model demonstrated good generalization ability in the internal validation cohort. Additionally, we identified the four most important clinical predictive features of infection after cardiac surgery: postoperative PLT, intraoperative autologous blood transfusion, postoperative AST, and postoperative WBC count. Several limitations also exist. First, as this was a retrospective study, there is potential for unexamined confounding factors and selection bias; however, we adopted the multicenter data may enhance the reliability of our results. The infection rate was consistent with each other in most hospitals. However, due to the small sample size in a few hospitals' data and lead to the Kruskal test value was not statistically meaningful (<0.05) (Supplementary File S9). Moreover, the reason for this discrepancy between hospitals each other was unknown and needed further study. Additionally, Unlike the traditional linear models, the entire machine learning process performs in a black box and lacks interpretability. And the external validation in our study was done on a very small number of cases. Another limitation is the lack of discrimination for the main outcome of interest which is infection. Our model is a dichotomous prediction model, which can only distinguish whether there is an infection but cannot predict the specific type of infection. We also generated prediction models based on our identified multiple variables for surgical site infection (SSI) and showed a good result. This result indicated that our identified multiple variables strongly correlated with surgical site infection. And it also indicated that there were some commonalities among the different types of infection. However, the relatively few SSI cases (15/1223), and further research was needed. Finally, this study focused on all infections occurring within 30 days postoperatively, and we did not collect information about when the infection happened. So, our model can only predict the infection or not infection without precisely the time of infection.

## Conclusion

The present study demonstrated the potential of machine learning algorithm-based methods for selecting features and generating postoperative infection-prediction tools. We identified critical perioperative variables and successfully established a machine-learning model to optimize infection risk prediction after mitral valve surgery. This approach could guide clinical treatment, decrease the risk of postoperative infection, and improve the prognosis of patients.

## Data Availability

The original contributions presented in the study are included in the article/**Supplementary Material**, further inquiries can be directed to the corresponding authors.

## References

[1] Pelosi P Ball L Schultz MJ. How to optimize critical care resources in surgical patients: intensive care without physical borders. Curr Opin Crit Care. (2018) 24(6):581–7. 10.1097/MCC.0000000000000557 30299312

[2] Dixon B Santamaria JD Reid D Collins M Rechnitzer T Newcomb AE The association of blood transfusion with mortality after cardiac surgery: cause or confounding? (CME). Transfusion. (2013) 53(1):19–27. 10.1111/j.1537-2995.2012.03697.x 22574710

[3] Ghaferi AA Birkmeyer JD Dimick JB. Complications, failure to rescue, and mortality with major inpatient surgery in medicare patients. Ann Surg. (2009) 250(6):1029–34. 10.1097/sla.0b013e3181bef697 19953723

[4] Wang TKM Akyuz K Kirincich J Duran Crane A Mentias A Xu B Comparison of risk scores for predicting outcomes after isolated tricuspid valve surgery. J Card Surg. (2022) 37(1):126–34. 10.1111/jocs.16098 34672020

[5] Gelijns AC Moskowitz AJ Acker MA Argenziano M Geller NL Puskas JD Management practices and major infections after cardiac surgery. J Am Coll Cardiol. (2014) 29:64(4):372–81. 10.1016/j.jacc.2014.04.052 PMC4222509 25060372

[6] Hajjar LA Vincent JL Galas FR Nakamura RE Silva CM Santos MH Transfusion requirements after cardiac surgery: the TRACS randomized controlled trial. JAMA. (2010) 13:304(14):1559–67. 10.1001/jama.2010.1446 20940381

[7] Lola I Levidiotou S Petrou A Arnaoutoglou H Apostolakis E Papadopoulos GS. Are there independent predisposing factors for postoperative infections following open heart surgery? J Cardiothorac Surg. (2011) 6:151. Published 2011 Nov 14. 10.1186/1749-8090-6-151 22082355 PMC3223138

[8] de la Varga-Martínez O Gómez-Sánchez E Muñoz MF Lorenzo M Gómez-Pesquera E Poves-Álvarez R Impact of nosocomial infections on patient mortality following cardiac surgery. J Clin Anesth. (2021) 69:110104. 10.1016/j.jclinane.2020.110104 33221707

[9] Fowler VJ O'Brien SM Muhlbaier LH Corey GR Ferguson TB Peterson ED. Clinical predictors of major infections after cardiac surgery. Circulation. (2005) 112(9 Suppl):I358–65. 10.1161/CIRCULATIONAHA.104.525790 16159846

[10] Abboud CS Wey SB Baltar VT. Risk factors for mediastinitis after cardiac surgery. Ann Thorac Surg. (2004) 77(2):676–83. 10.1016/S0003-4975(03)01523-6 14759458

[11] Pronovost P Needham D Berenholtz S Sinopoli D Chu H Cosgrove S An intervention to decrease catheter-related bloodstream infections in the ICU. N Engl J Med. (2006) 28:355(26):2725–32. 10.1056/NEJMoa061115 17192537

[12] Bode LG Kluytmans JA Wertheim HF Bogaers D Vandenbroucke-Grauls CM Roosendaal R Preventing surgical-site infections in nasal carriers of Staphylococcus aureus. N Engl J Med. (2010) 7:362(1):9–17. 10.1056/NEJMoa0808939 20054045

[13] Horvath KA Acker MA Chang H Bagiella E Smith PK Iribarne A Blood transfusion and infection after cardiac surgery. Ann Thorac Surg. (2013) 95(6):2194–201. 10.1016/j.athoracsur.2012.11.078 23647857 PMC3992887

[14] Esteva A Kuprel B Novoa RA Ko J Swetter SM Blau HM Dermatologist-level classification of skin cancer with deep neural networks. Nature. (2017) 2:542(7639):115–18. 10.1038/nature21056 28117445 PMC8382232

[15] Shickel B Tighe PJ Bihorac A Rashidi P Deep EHR. A survey of recent advances in deep learning techniques for electronic health record (EHR) analysis. IEEE J Biomed Health Inform. (2018) 22(5):1589–604. 10.1109/JBHI.2017.2767063 29989977 PMC6043423

[16] Remenyi B ElGuindy A Smith SJ Yacoub M Holmes DJ. Valvular aspects of rheumatic heart disease. Lancet. (2016) 387(10025):1335–46. 10.1016/S0140-6736(16)00547-X 27025439

[17] Westphal S Stoppe C Gruenewald M Bein B Renner J Cremer J Genome-wide association study of myocardial infarction, atrial fibrillation, acute stroke, acute kidney injury and delirium after cardiac surgery - a sub-analysis of the RIPHeart-Study. BMC Cardiovasc Disord. (2019) 24:19(1):26. 10.1186/s12872-019-1002-x PMC6345037 30678657

[18] Bodenhofer U Haslinger-Eisterer B Minichmayer A Hermanutz G Meier J. Machine learning-based risk profile classification of patients undergoing elective heart valve surgery. Eur J Cardiothorac Surg. (2021) 60(6):1378–85. 10.1093/ejcts/ezab219 34050368

[19] Dhippayom T Dilokthornsakul P Laophokhin V Kitikannakorn N Chaiyakunapruk N. Clinical burden associated with postsurgical complications in major cardiac surgeries in Asia-oceania countries: a systematic review and meta-analysis. J Card Surg. (2020) 35(10):2618–26. 10.1111/jocs.14855 32743909

[20] Ridgway ZA Howell SJ. Cardiopulmonary exercise testing: a review of methods and applications in surgical patients. Eur J Anaesthesiol. (2010) 27(10):858–65. 10.1097/EJA.0b013e32833c5b05 20689441

[21] Vesteinsdottir E Helgason KO Sverrisson KO Gudlaugsson O Karason S. Infections and outcomes after cardiac surgery-the impact of outbreaks traced to transesophageal echocardiography probes. Acta Anaesthesiol Scand. (2019) 63(7):871–8. 10.1111/aas.13360 30888057 PMC6619098

[22] McClure GR Belley-Cote EP Harlock J Lamy A Stacey M Devereaux PJ Steroids in cardiac surgery trial: a substudy of surgical site infections. Can J Anaesth. (2019) 66(2):182–92. English. 10.1007/s12630-018-1253-5 30535668

[23] Mocanu V Buth KJ Johnston LB Davis I Hirsch GM Légaré JF. The importance of continued quality improvement efforts in monitoring hospital-acquired infection rates: a cardiac surgery experience. Ann Thorac Surg. (2015) 99(6):2061–9. 10.1016/j.athoracsur.2014.12.075 25795297

[24] Hortal J Muñoz P Cuerpo G Litvan H Rosseel PM Bouza E Ventilator-associated pneumonia in patients undergoing major heart surgery: an incidence study in Europe. Crit Care. (2009) 13(3):R80. 10.1186/cc7896 19463176 PMC2717444

[25] Segers P Speekenbrink RG Ubbink DT van Ogtrop ML de Mol BA. Prevention of nosocomial infection in cardiac surgery by decontamination of the nasopharynx and oropharynx with chlorhexidine gluconate: a randomized controlled trial. JAMA. (2006) 296(20):2460–6. 10.1001/jama.296.20.2460 17119142

[26] Wan YI Patel A Abbott TEF Achary C MacDonald N Duceppe E Prospective observational study of postoperative infection and outcomes after noncardiac surgery: analysis of prospective data from the VISION cohort. Br J Anaesth. (2020) 125(1):87–97. 10.1016/j.bja.2020.03.027 32482502

[27] Ellis DE Hubbard RA Willis AW Zuppa AF Zaoutis TE Hennessy S. Comparing LASSO and random forest models for predicting neurological dysfunction among fluoroquinolone users. Pharmacoepidemiol Drug Saf. (2022) 31(4):393–403. 10.1002/pds.5391 34881470

[28] Hu P Liu Y Li Y Guo G Su Z Gao X A Comparison of LASSO Regression and Tree-Based Models for Delayed Cerebral Ischemia in Elderly Patients With Subarachnoid Hemorrhage. Front Neurol. (2022) 10:13:791547. 10.3389/fneur.2022.791547 PMC8960268 35359648

[29] Meng L Zheng T Wang Y Li Z Xiao Q He J Development of a prediction model based on LASSO regression to evaluate the risk of non-sentinel lymph node metastasis in Chinese breast cancer patients with 1-2 positive sentinel lymph nodes. Sci Rep. (2021) 7:11(1):19972. 10.1038/s41598-021-99522-3 PMC8497590 34620978

[30] Vermeijden WJ van Klarenbosch J Gu YJ Mariani MA Buhre WF Scheeren TW Effects of cell-saving devices and filters on transfusion in cardiac surgery: a multicenter randomized study. Ann Thorac Surg. (2015) 99(1):26–32. 10.1016/j.athoracsur.2014.08.027 25440265

[31] Wang G Bainbridge D Martin J Cheng D. The efficacy of an intraoperative cell saver during cardiac surgery: a meta-analysis of randomized trials. Anesth Analg. (2009) 109(2):320–30. 10.1213/ane.0b013e3181aa084c 19608798

[32] van Klarenbosch J van den Heuvel ER van Oeveren W de Vries AJ. Does intraoperative cell salvage reduce postoperative infection rates in cardiac surgery? J Cardiothorac Vasc Anesth. (2020) 34(6):1457–63. 10.1053/j.jvca.2020.01.023 32144053

[33] Mahmood E Knio ZO Mahmood F Amir R Shahul S Mahmood B Preoperative asymptomatic leukocytosis and postoperative outcome in cardiac surgery patients. PLoS One. (2017) 5:12(9):e0182118. 10.1371/journal.pone.0182118 PMC5584953 28873411

[34] Heredia-Rodríguez M Bustamante-Munguira J Lorenzo M Gómez-Sánchez E Álvarez FJ Fierro I Procalcitonin and white blood cells, combined predictors of infection in cardiac surgery patients. J Surg Res. (2017) 5:212:187–94. 10.1016/j.jss.2017.01.021 28550906

[35] Li J Li R Jin X Ren J Du L Zhang J Association of platelet count with mortality in patients with infectious diseases in intensive care unit: a multicenter retrospective cohort study. Platelets. (2022) 17:33(8):1168–74. 10.1080/09537104.2022.2066646 35485162

[36] Aloisio E Colombo G Arrigo C Dolci A Panteghini M. Sources and clinical significance of aspartate aminotransferase increases in COVID-19. Clin Chim Acta. (2021) 522:88–95. 10.1016/j.cca.2021.08.012 34411557 PMC8366047

[37] Hu Q Zhao Y Sun B Qi W Shi P. Surgical site infection following operative treatment of open fracture: incidence and prognostic risk factors. Int Wound J. (2020) 17(3):708–15. 10.1111/iwj.13330 32068337 PMC7949428

