# Supplementary material for: Identification of risk factors for infection after mitral valve surgery through machine learning approaches
Source: Front Cardiovasc Med. 2023 Jun 13;10:1050698. doi: 10.3389/fcvm.2023.1050698 (PMC10294678; doi:10.3389/fcvm.2023.1050698)
Supplement: Supplementary file 1 [file Datasheet1.pdf]

Supplementary File 1 Variables screened by machine learning algorithms (RF and LASSO)

ML method	variables
RF	col44; col30; col56; col36; col22; col58; col27; col52; col28; col60; col57; col32; col46; col59; col48; col50; col33; col64; col78; col54; col63; col49; col43; col40; col8; col6; col42; col21; col35; col19; col45; col47; col20; col51; col77; col37; col67; col38; col53; col4; col34; col39; col55; col9; col26; col17; col70
LASSO	col3; col4; col6; col8; col10; col11; col13; col14; col17; col18; col21; col27; col28; col30; col33; col36; col39; col43; col44; col45; col54; col56; col57; col63; col64; col65; col68; col69; col72; col77; col78; col84; col85; col92; col93

Col3: gender; col4: age; col6: weight; col8: stay of hospital; col9: ICU time; col11: LV dilatation; col13: Diabetes; col14: anemia; col16: Cerebrovascular disease; col17: NYHA; col18: ASA; col19: surgery time; col20: CBP time; col21: ACC time; col22: Cardiopulmonary bypass precharge; col26: urine output; col27: total output; col28: total input; col30: Autologous blood; col32: minimum oxygen saturation; col33: pre RBC; col34: post RBC; col35: pre WBC; col36: post WBC; col37: pre Hb; col38: intraoperative minimum Hb; col39:post Hb; col40: pre Hct; col42: post Hct; col43: pre PLT; col44: post PLT; col45: pre Cr; col46: post Cr; col47: pre TP; col48: post TP; col49: pre albumin; col50: post albumin; col51: pre globulin; col52: post globulin; col53: pre ALT; col54: post ALT; col55: pre AST; col56: post AST; col57: pre PT; col58: post PT; col59: pre INR; col60: post INR; col63: per LVEF; col64: post LVEF; col65: pre RBC transfusion; col67: post RBC transfusion; col68: pre FFP transfusion; col:69: intraoperative FFP transfusion; col70: post FFP transfusion; col72: intraoperative PLT transfusion; col77: total RBC transfusion; col78: total FFP transfusion; col84: In-hospital deaths; col85: postoperative myocardial infarction; col92: postoperative Low cardiac output sysdrome; col93: postoperative mitral regurgitation

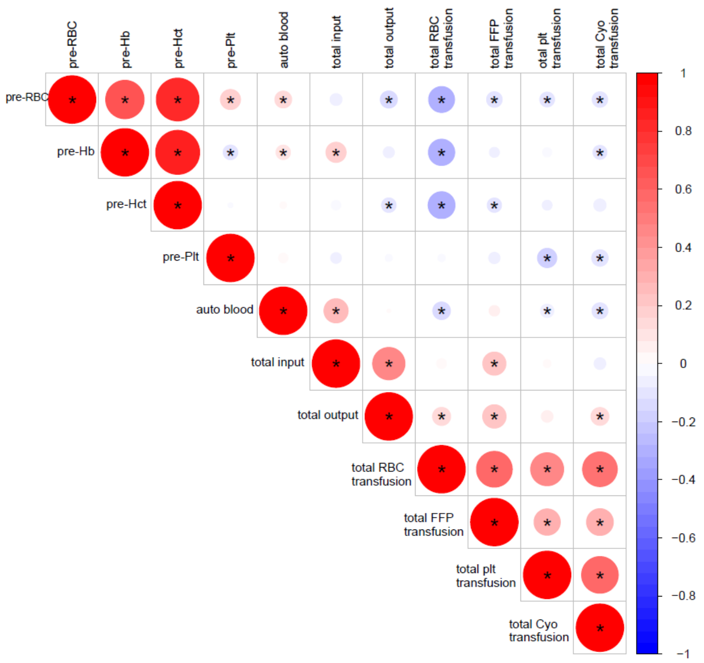

Supplementary File 2. The correlation by heat map analysis. The X axis and Y axis represent the selected variables and the color represent the correlation, where low values are blue, and high values are red.

Supplemental File 3 Compared the pairwise performance of ML models in Test set

	RF	SVM	xgboost	GBDT	adaboost	NB	LogicR	nnet	ANN	Mcnemar's Test P-Value
RF	NA	0.2888	0.0736	0.0736	0.1824	0.0000	0.0872	0.0771	0.0039	0.0718
SVM	NA	NA	1.0000	1.0000	1.0000	1.0000	0.0327	0.6171	0.0119	0.1878
xgboost	NA	NA	NA	NA	1.0000	0.0000	0.0251	1.0000	0.0258	0.2188
GBDT	NA	NA	NA	NA	1.0000	0.0000	0.0251	1.0000	0.0258	0.2188
adaboost	NA	NA	NA	NA	NA	0.0000	0.0251	1.0000	0.0176	0.2188
NB	NA	NA	NA	NA	NA	NA	0.0250	0.0000	0.0000	0.0000
LogicR	NA	NA	NA	NA	NA	NA	NA	0.0192	0.0003	0.0040
nnet	NA	NA	NA	NA	NA	NA	NA	NA	0.0367	0.2665
ANN	NA	NA	NA	NA	NA	NA	NA	NA	NA	0.9151

Significance of differences $p < 0.05$

Supplementary File 4: The assessment parameters of ANN model.

	Non-infection	Infection	Accuracy	Precision	Sensitivity	Specificity	Positive Predictive Value	Negative Predictive Value
Train non-infection	508	88	0.903738	0.671642	0.692308	0.852349	67.16418	86.39456
infection	80	180						
Test non-infection	220	45	0.758904	0.57	0.558824	0.836502	57	83.01887
infection	43	57						

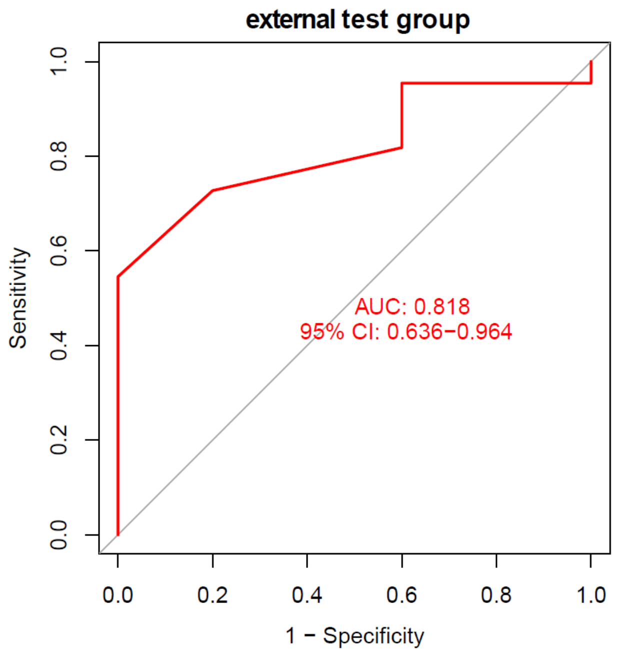

Supplementary File 5. The area under the ROC curve (ROC area) of external validation

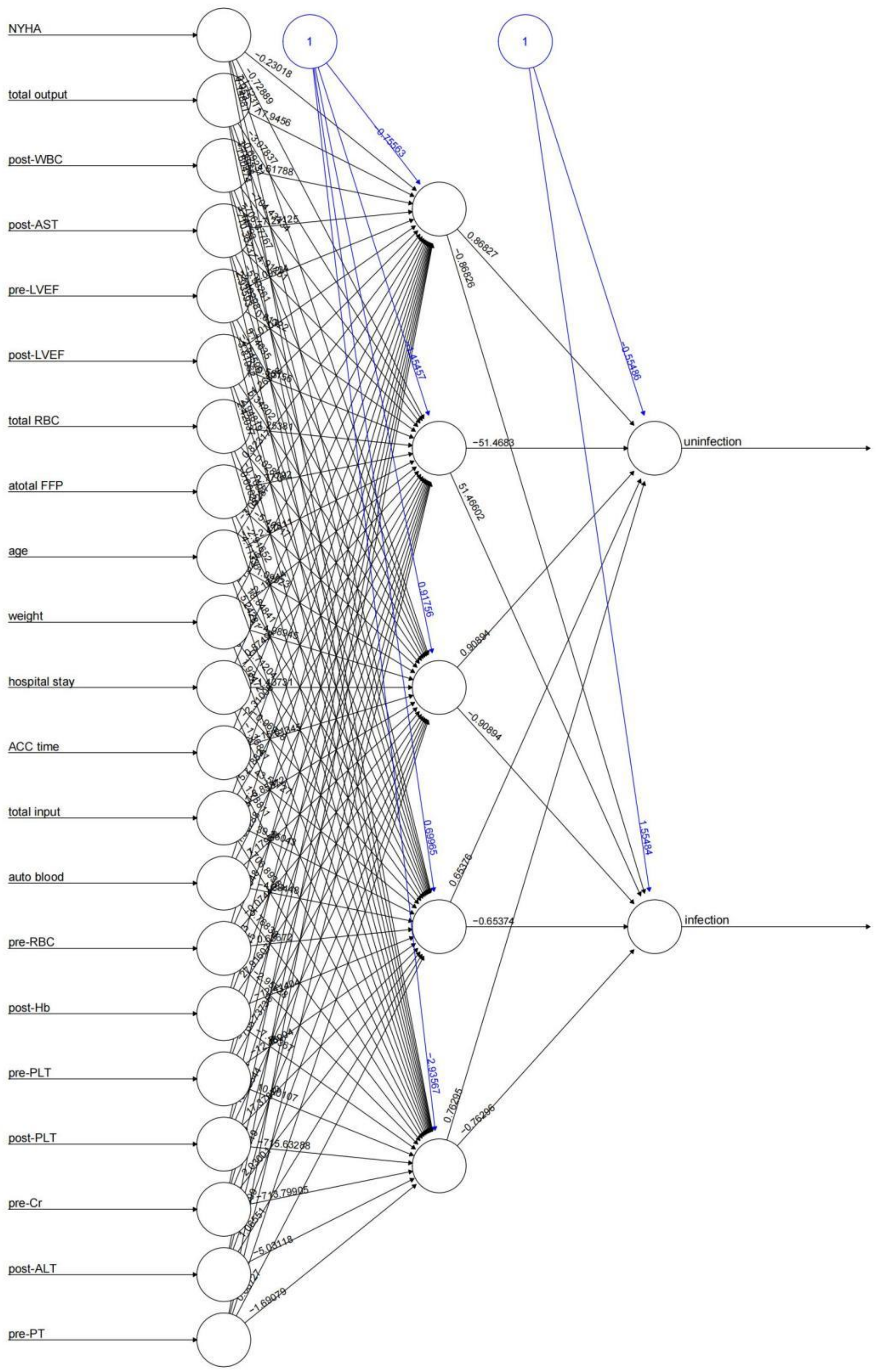

Supplementary File 6. ANN model with parameters

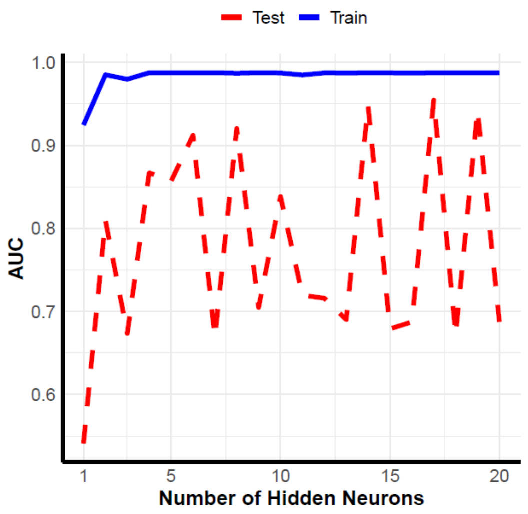

Supplementary File 7. Validation curve for ANN model

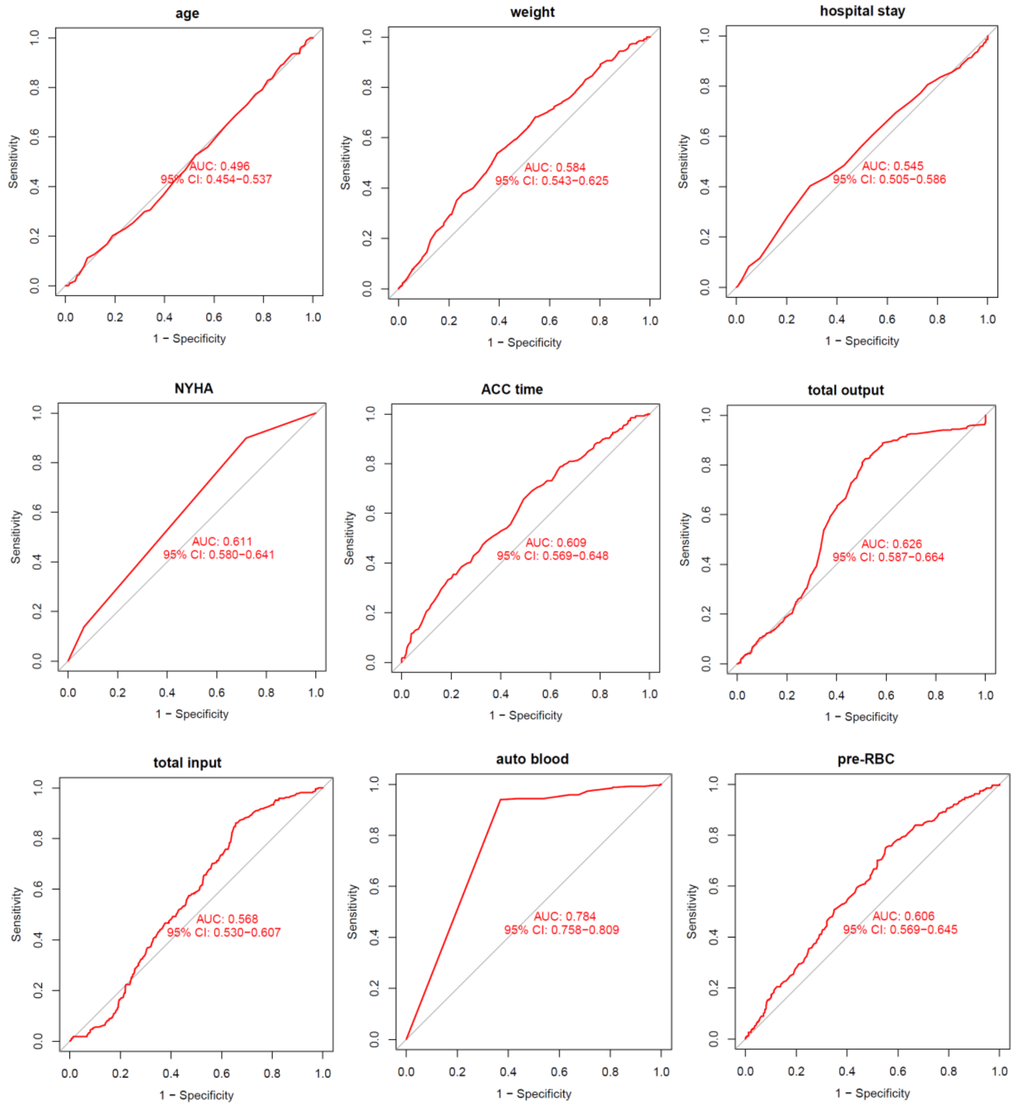

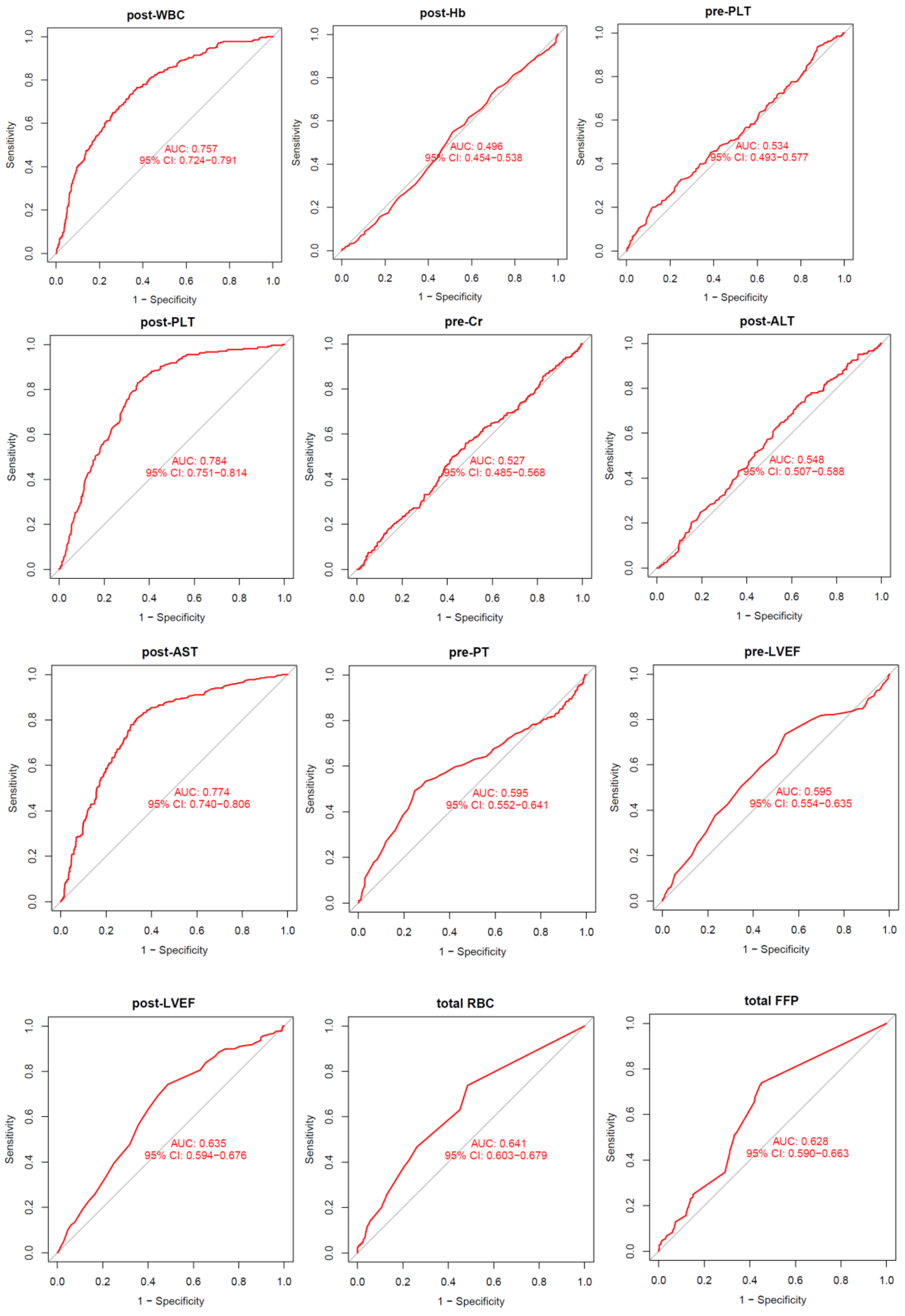

Supplementary File 8. The area under the ROC curve (ROC area) for each variable

Supplementary File 9 The infection rate at each hospital

Hospital	Infection rate(n, %)
Fuwai Hospital National Center for Cardiovascular Diseases	45/278, 16.2
Beijing Aerospace General Hospital	0/2, 0
Qilu Hospital of Shandong University	25/167, 15.0
Affiliated Hospital of Southwest Medical University	21/126, 16.7
Zhejiang Provincial People's Hospital	12/24, 50.0
Xiamen Cardiovascular Hospital	29/184, 15.8
The Third Xiangya Hospital of Central South University	16/16, 100
The Second Xiangya Hospital of Central South University	220/426, 51.6
